# Supplementary material for: Population interest in and adequacy of the information on the safety of antineoplastic agents in the Spanish edition of Wikipedia
Source: PLoS One. 2025 Apr 22;20(4):e0320773. doi: 10.1371/journal.pone.0320773 (PMC12013910; doi:10.1371/journal.pone.0320773)
Supplement: Attachment — Submitted filename: Response to Reviewers.docx [file pone.0320773.s002.docx]

**Point-by-point response to the reviewer’s comments**

The authors thank the corrections and comments made. Suggestions for improvement provided by reviewers and editorial, have seemed us to be very appropriate and useful. We are confident that they have contributed to improving our article.

All modifications or additions made are listed in this document and are brightly-colored in the text of the manuscript for your easy viewing.

**Revisions carried out**

**Reviewer #1**

The initiative of the study is interesting in terms of therapeutic education but methodologically the work can be improved.

The fact that the study measures a negative correlation between the use of anticancer drugs and visits to Wikipedia pages does not seem enlightening to me. It is enough that over the years the referencing of websites changes for other 'Vidal' type websites to appear first in the results of the 'google' type web search before Wikipedia. So naturally Wikipedia will be less consulted.

- Indeed, the results may change over the years and, therefore, this study should be updated. However, when selecting the Web 2.0 reference, we took into account the current results, as indicated in references 11 and 12, where we can find Wikipedia in position 7 in Similarweb and in position 5 in Semrush.
- On the other hand, we could have studied the results offered by Google Trends, but these are offered in percentage of search (relative search volume) with respect to the maximum search volume at a given time and, consequently, the relative search volume will fluctuate depending on the period studied. Consequently, it was decided to use the data from Wikipedia as this is real data on the number of searches and does not vary.
- As we consider that this explanation may be interesting, in accordance with the reviewer's warning, we have included it in the limitations of the study.

Then the application of regression models (the nature of which we do not know) and correlation on such a small number of observations does not seem appropriate to me.

- We have modified the data analysis section in the methodology, explaining better the use of linear regression models and we have added a sentence in the results explaining, also, the data of the association between the consumption of antineoplastic drugs and the queries made to their terms in the Spanish edition of the Wikipedia.

**Reviewer #2**

The manuscript was written on a very important topic where Web 2.0 became the main source of information in general, and the medical topics in specific. However, the authors have to check that mentioning of the compound term prior is abbreviation to avoid readers' misleading (example, "AA" in the abstract).

- We had forgotten to define the abbreviation AA in the abstract. However, taking advantage of the reviewer's recommendation, it is true that the abbreviation AA can lead to confusion as it is the abbreviation generally used for acetylsalicylic acid. On the other hand, neither the FDA (Food and Drug Administration) nor the EMA (European Medicines Agency) use a universally standardised abbreviation for antineoplastic agents. However, a review of the scientific literature has shown that ANP is the most commonly used abbreviation. We have therefore changed the abbreviation AA to ANP.

Introduction section: There must be indication for recent data from IARC, as a legitimate source of updated statistics, where the number of neoplasms-related deaths became 10 million in 2022 (Refer to: https://www.iarc.who.int/news-events/new-report-on-global-cancer-burden-in-2022-by-world-region-and-human-development-level/) and the authors must avoid the use of referral references.

Besides, several paragraphs at the introduction section have no referral to any citated references, and thus the quality of those discussed information is questionable (paragraphs 2,5 and 9)

- The first paragraph of the introduction has been modified taking into account the bibliographical citation provided by the reviewer.
- References have been added to the paragraphs indicated by the reviewer.

Materials and Methods: the number of topics being collected among popular Wikipedia pages were missing.

- In the section ‘ANP and Wikipedia pages studied’, the topics studied have been specified both in terms of consumption and in Wikipedia.

The ethical aspects must be declared at first prior going into further details of the study methodology.

- The section on ethical issues has been placed at the beginning of the methodology, after the study design, as requested by the reviewer.

The data quality as well as the statistical analysis must be performed by at least two authors / researchers in a blinded data selection, and handling the statistical analysis va validated software (as SPSS, STATA, R studio, SAS) was not clearly discussed in depth.

- How the data was collected and the correction of discrepancies has been included in the ‘Data analysis’ section.

However, the authors must provide further information regarding how CIMA information center can be used as legitimate source and what type of validity being applied in the center to ensure its data accuracy.

- CIMA is the database of medicines produced by the Spanish Agency of Medicines and Health Products (AEMPS). The AEMPS as a state agency attached to the Ministry of Health is a reference entity in Spain in charge of protecting public health through the strict regulation of medicines and health products. The AEMPS is considered highly reliable within the Spanish and European healthcare system as it operates under strict national and European regulations, and its procedures are aligned with the standards of the European Medicines Agency (EMA). Therefore, its data have a high degree of reliability.
- A paragraph explaining CIMA's reliability has been included in the methodology.

Discussion: According to FDA Pregnancy category and drugs -related risks information, generally chemotherapy and AA in specific are considered category X medication; where studies in animals or humans have demonstrated positive evidence of human fetal risk based on adverse reaction data from investigational or marketing experience, and the risks involved in use of the drug in pregnant women clearly outweigh potential benefits. Thus, no such information regarding the use of AA in pregnant and breast feeding, or its impact on infants exposure.

- Indeed, there is no information on the use of antineoplastic agents in pregnant and breastfeeding women, nor on their impact on infant exposure, since being category X the risk clearly outweighs the possible benefits, they are not used = no data. To further clarify this information, we have expanded the paragraph in the discussion.
