# Supplementary material for: Population interest in and adequacy of the information on the safety of antineoplastic agents in the Spanish edition of Wikipedia
Source: PLoS One. 2025 Apr 22;20(4):e0320773. doi: 10.1371/journal.pone.0320773 (PMC12013910; doi:10.1371/journal.pone.0320773)
Supplement: Attachment — Submitted filename: Response to Reviewers - MR.docx [file pone.0320773.s003.docx]

**Point-by-point response to the reviewer’s comments**

First of all, we would like to thank you for the new review of our manuscript.

All changes made to this document are highlighted in bright colors in the text of the manuscript so you can easily see them.

**Revisions carried out**

All bibliographic references have been thoroughly reviewed, and no errors have been found nor have any retracted articles been cited.

**Reviewer #2**

I think that the authors have fulfilled and well-explained all peer-reviewers remarks and notes on the basis of clinical and scientific tracks, and made all the recommended corrections punctually.

However, some efforts to change the abbreviation AA to ANP in the abstract section (at "Mansucript draft; page # 1) are required as being applied on the whole manuscript.

- In accordance with the reviewer's comments, all abbreviations 'AA' have been corrected and replaced with 'ANP'. Previously, they were highlighted in yellow, and in this latest review, they have been highlighted in green.
